# The Role of FtsH Complexes in the Response to Abiotic Stress in Cyanobacteria

**DOI:** 10.1093/pcp/pcae042

**Published:** 2024-04-15

**Authors:** Vendula Krynická, Josef Komenda

**Affiliations:** Institute of Microbiology of the Czech Academy of Sciences, Centre Algatech, Opatovický Mlýn, Novohradská 237, Třeboň 37901, The Czech Republic; Institute of Microbiology of the Czech Academy of Sciences, Centre Algatech, Opatovický Mlýn, Novohradská 237, Třeboň 37901, The Czech Republic

**Keywords:** Cyanobacteria, FtsH, Nutrient stress, Photodamage, Photosystem

## Abstract

FtsH proteases (FtsHs) belong to intramembrane ATP-dependent metalloproteases which are widely distributed in eubacteria, mitochondria and chloroplasts. The best-studied roles of FtsH in *Escherichia coli* include quality control of membrane proteins, regulation of response to heat shock, superoxide stress and viral infection, and control of lipopolysaccharide biosynthesis. While heterotrophic bacteria mostly contain a single indispensable FtsH complex, photosynthetic cyanobacteria usually contain three FtsH complexes: two heterocomplexes and one homocomplex. The essential cytoplasmic FtsH1/3 most probably fulfills a role similar to other bacterial FtsHs, whereas the thylakoid FtsH2/3 heterocomplex and FtsH4 homocomplex appear to maintain the photosynthetic apparatus of cyanobacteria and optimize its functionality. Moreover, recent studies suggest the involvement of all FtsH proteases in a complex response to nutrient stresses. In this review, we aim to comprehensively evaluate the functions of the cyanobacterial FtsHs specifically under stress conditions with emphasis on nutrient deficiency and high irradiance. We also point to various unresolved issues concerning FtsH functions, which deserve further attention.

## Introduction

Acclimation to abiotic stress is a fundamental survival strategy employed by a wide range of organisms, spanning the entire biological spectrum. Cyanobacteria, a group of bacteria performing oxygenic photosynthesis, have developed intricate mechanisms to effectively cope with abiotic stress, primarily through precise regulation of gene expression and proteostasis. Diverse regulatory pathways contribute to these responses, including two-component systems, transcription factors and post-transcriptional control mediated by small regulatory sRNAs. While the activation of genes results in the synthesis and accumulation of new proteins important for the effective response, other proteins, frequently products of suppressed genes or damaged proteins, need to be actively removed under stress conditions. All these processes are governed by regulatory proteins whose concentrations require precise regulation, and the coordinated interaction of such pathways plays a crucial role in maintaining metabolic homeostasis.

The transmembrane FtsH metalloproteases (FtsHs) found universally in bacteria, chloroplasts and mitochondria (Ito and Akiyama 2005, Adam et al. 2006), play a crucial role in the quality control of membrane proteins through the degradation of misfolded or damaged proteins. Additionally, they significantly contribute to adaptive processes by modulating the levels of various regulatory proteins, such as transcription factors (Arends et al. 2016, Krynická et al. 2019, Yeo et al. 2020). Consequently, they are indispensable for preserving the stability of cellular functions in the majority of bacterial species. However, the precise molecular mechanisms underpinning the FtsH requirements remain largely elusive and a number of known FtsH substrates also remain rather limited. Most bacteria possess a single *ftsH* gene that encodes a single protein-forming hexameric homocomplexes with diverse housekeeping roles (Langklotz et al. 2012). However, in mitochondria, chloroplasts and cyanobacteria, multiple FtsH homologs form homo- and heterocomplexes with specialized functions in maintaining photosynthesis and respiration (Janska et al. 2013, Shao et al. 2018).

Most cyanobacteria, including *Synechocystis* sp. PCC 6803 (hereafter *Synechocystis*), possess four FtsH homologs, namely, FtsH1 to FtsH4 (Mann et al. 2000, Silva et al. 2003). However, numbering by homology is unfortunately not uniform within cyanobacteria. In this review, we will use the numbering of homologs according to *Synechocystis*. These homologs assemble into three oligomeric complexes in vivo: two hetero-oligomers FtsH1/3, FtsH2/3 and one homo-oligomer FtsH4 (Boehm et al. 2012). The least abundant FtsH1/3 complex (Jackson et al. 2023) is primarily situated in the plasma membrane (PM) (Krynická et al. 2014, Sacharz et al. 2015), while the more abundant FtsH2/3 and FtsH4 are localized in the thylakoid membranes (TMs) (Boehm et al. 2012). Based on *Arabidopsis* nomenclature, both FtsH1 and FtsH2 are categorized as type B FtsH (Sakamoto 2003, Bečková et al. 2017b) and share the highest sequence identity despite different cellular localizations, while their partner FtsH3 resembles the type A FtsH subunits of *Arabidopsis*. FtsH4, on the other hand, belongs to a later evolutionary group together with *Arabidopsis* FtsH homologs AtFtsH7 and AtFtsH9. However, while FtsH4 is located in cyanobacterial thylakoids, AtFtsH7 and AtFtsH9 were detected in the chloroplast envelope (Ferro et al. 2003, 2010). There are no data about the substrate specificity of these chloroplast proteases, and it is possible that during evolution, this specificity and related function diversified from those of FtsH4. Interestingly, among cyanobacterial strains with the incomplete set of aforementioned FtsH paralogs, the most frequently missing version is FtsH4 (Shao et al. 2018), indicating its dispensability.

Unlike the other two complexes, FtsH1/3 is essential for cell viability (Mann et al. 2000), although the underlying reasons remain elusive. In *Escherichia coli* (E. coli), FtsH plays a vital role in quality control of membrane proteins, in regulation of the response to heat stress and viral infection and in maintaining the lipopolysaccharide/phospholipid ratio through the turnover of LpxC deacetylase (Ogura et al. 1999). Similar functions can be expected but are not yet proven for FtsH1/3. Till now, it has been shown that *Synechocystis* FtsH1/3 is crucial for fine-tuning the transcriptional nutrient stress response in *Synechocystis* (Krynická et al. 2019). On the other hand, the thylakoid FtsHs are more implicated in photosynthetic processes. FtsH2/3 governs the selective degradation of photosystem II (PSII) subunits during PSII repair, playing a crucial role in maintaining active PSII complexes (Silva et al. 2003, Komenda et al. 2006). Deletion of the *ftsH2* gene leads to light sensitivity and photoinhibition of PSII and thus affects autotrophic growth already under normal light (NL) conditions. Additionally, its phenotype involves a reduction in the photosystem I (PSI) level and chlorophyll (Chl) content of the cell (Mann et al. 2000). The other thylakoid FtsH4 is engaged in the biogenesis of PSII and PSI and acclimation to sudden changes in light intensity (Krynická et al. 2023, Koník et al. 2024).

## Acclimation to Nutrient Stress

**Fig. 1:**
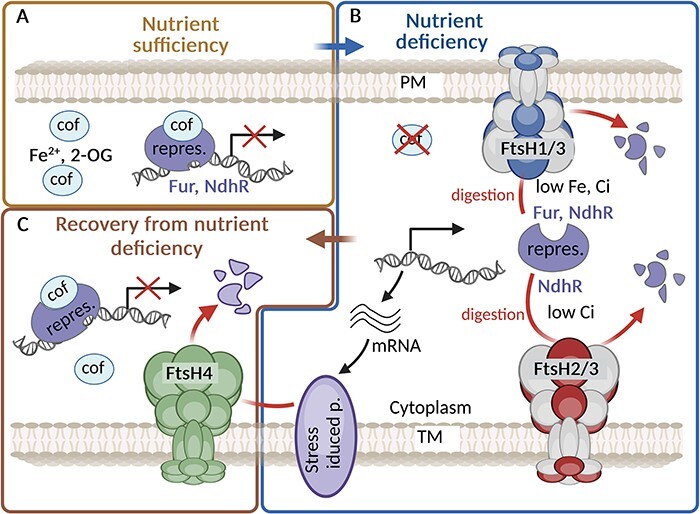
The role of FtsH complexes in maintaining iron or inorganic carbon homeostasis. (A) Under iron (Fe)/inorganic carbon (Ci) sufficiency, the repressor Fur/NdhR binds the corresponding cofactor (cof) Fe^2+^/2-oxoglutarate (2-OG), which increases its affinity to DNA, and this leads to repression of the relevant regulon. (B) Under Fe/Ci deficiency, the amount of cof is reduced, which leads to a release of the repressors from the DNA and hence to expression of the relevant regulons. The level of both Fur and NdhR repressors released from DNA binding to cytoplasm is controlled by FtsH1/3 and FtsH2/3, respectively. (C) After regaining nutrients, FtsH4 helps recovery from stress by degrading Fur-regulated proteins such as IsiA and putatively also NdhR-regulated proteins participating in CCM. Created using BioRender.com.

**Fig. 2:**
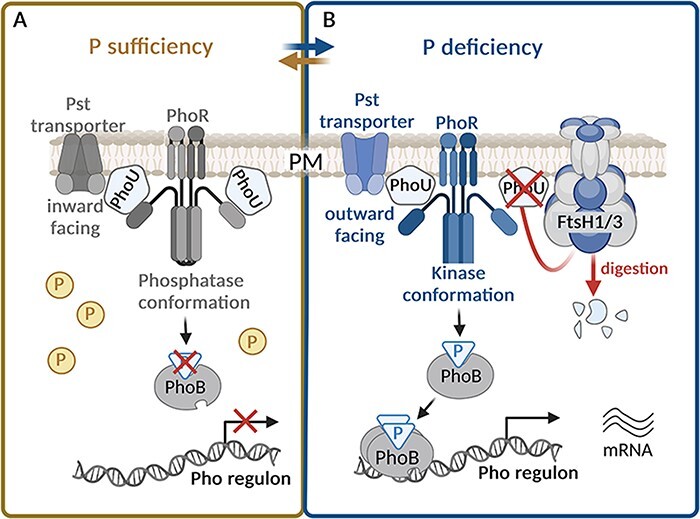
The role of FtsH1/3 in the fast acclimation to phosphorus deficiency. (A) Under phosphorus (P) sufficiency, the PhoU-negative regulator accumulates in the cell (Krynická et al. 2019). PhoU binds to the Pst transporter, and consequently, the transporter is present in the closed (inward facing) conformation (McCleary 2017), so the P transport is stopped. At the same time, PhoU interacts with PhoR and stabilizes it in the phosphatase conformation (McCleary 2017). Consequently, the PhoR dephosphorylates the response regulator PhoB and the Pho regulon is silent. (B) Under P deficiency, the level of PhoU is reduced by FtsH1/3 (Krynická et al. 2019). PhoU does not interact with PhoR, which consequently switches to kinase conformation. It leads to phosphorylation of PhoB, which increases its binding affinity to DNA. PhoB thus activates expression of Pho regulon, which leads to the synthesis of the Pst transporters. At the same time, those transporters switch from a position facing inward to outward and pump the P from the periplasm to the cytoplasm (McCleary 2017). Created using BioRender.com.

**Fig. 3:**
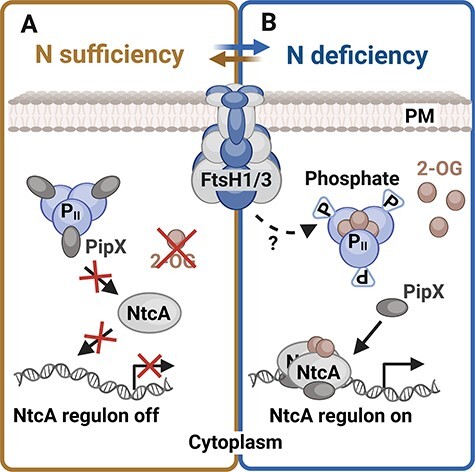
The role of the FtsH1/3 complex in the activation of the NtcA regulon. (A) Under N sufficiency, the level of 2-OG (2-oxoglutarate) is low and the PipX protein is bound in a complex with the P_II_ regulatory protein. Therefore, the affinity of NtcA transcription factor to DNA is low, and NtcA regulon is silent (Espinosa et al. 2014). (B) Under N-deplete conditions and high C/N ratio, 2-OG accumulates in the cell. P_II_ protein is saturated with 2-OG and becomes phosphorylated. P_II_ phosphorylation is facilitated by FtsH1/3 via an unknown mechanism. Consequently, the PipX protein dissociates from P_II_ and binds together with 2-OG to NtcA. Formation of the NtcA/2-OG/PipX complex increases the affinity of NtcA transcription factor to DNA, which leads to induction of NtcA regulon response. Created using BioRender.com.

Several studies have demonstrated that FtsHs ultimately influence the ability to acclimate to nutrient depletion in *Synechocystis* (Zhang et al. 2007, Krynická et al. 2014, 2019). Under nutrient-limited conditions, cyanobacteria undergo significant changes in gene expression to adapt to the limited availability of the corresponding nutrients. The FtsHs, especially FtsH1/3, facilitate quick transcription responses of specific regulons under iron (Fe), phosphorus (P), carbon (C), specifically inorganic carbon (Ci), and nitrogen (N) limitation by modulating the level of regulatory proteins such as transcription repressors. In FtsH1/3-depleted cells, these repressors accumulate during nutritional stress, resulting in a slower transcriptional response to stress and persistent nutritional imbalance (Krynická et al. 2019). The role of FtsH complexes in acclimation to nutritional stress is schematically illustrated in **Figs. 1**–**3**.

### The key role of FtsH in adaptation to fluctuating Fe levels

The role of the *Synechocystis* FtsH1/3 complex in orchestrating an efficient stress response has been most extensively investigated under Fe depletion (Krynická et al. 2014, 2019). Fe depletion in cyanobacteria triggers a series of processes that impact the photosynthetic activity and the expression of specific proteins, especially the Fe stress-induced Chl-binding protein A (IsiA). IsiA forms a giant ring structure around PSI and acts as a major Chl-containing protein in Fe-starved cyanobacteria (Dühring et al. 2006) optimizing the light-harvesting capacity of the complex [reviewed in Jia et al. (2021)]. Apart from IsiA, Fe depletion induces the expression of proteins associated with Fe acquisition, storage and utilization. The transcriptional regulation of these genes in cyanobacteria is primarily governed by the transcription factor called Ferric uptake regulator (Fur) (Gonzalez et al. 2014, 2016). Fur is a global transcriptional regulator conserved in most prokaryotes (Fillat 2014). Under Fe-rich conditions, Fur predominantly serves as a repressor by binding to specific DNA sequences known as Fur boxes, thus inhibiting the transcription of target genes (Gonzalez et al. 2014, Riediger et al. 2021). However, under Fe limitation, the Fur loses Fe ion and its binding to DNA is consequently destabilized allowing the expression of Fur-controlled genes.

Downregulation of FtsH1/3 has been linked to the sustained suppression of Fur-regulated genes including *isiA* and Fe transporters. Remarkably, during Fe depletion in the *Synechocystis* wild type (WT) strain, the concentration of free Fur (i.e. the one not bound to DNA or membrane) drops significantly, whereas in the FtsH1/3 knock-down mutant, it remains stable (Krynická et al. 2014). This points to the fact that FtsH1/3 actively regulates the level of the ‘free’ Fur to prevent its reattachment to DNA (**Fig. 1**). Direct degradation of Fur by FtsH1/3 has also been confirmed in vitro (Krynická et al. 2019). In accordance with these findings, an excess of FtsH1/3 induces a response of the Fur regulon even under conditions of nutrient sufficiency, suggesting a potential Fur degradation even in the presence of Fe. Under Fe limitation, the lack of functional FtsH1/3 is additionally linked to the repression of the *sufBCDS* operon, crucial for iron–sulfur cluster biosynthesis (Shen et al. 2007, Krynická et al. 2019). Expression of this operon is induced specifically by Fe deficiency or oxidative stress. The SufR protein emerges as a key regulator in this context, functioning as a transcriptional repressor of the operon (Shen et al. 2007). Consequently, SufR may be considered another potential substrate for degradation by FtsH1/3.

Beyond their role in sustaining Fe homeostasis during Fe deficiency, FtsHs also play a critical role in recovery from this nutritional stress. During the recovery, IsiA levels are reduced, aiding the rapid restoration of the standard PSI level. Recent studies have revealed that FtsH4 is involved in IsiA degradation during the recovery process, which enables fast resuming of photosynthetic productivity and growth (Koník et al. 2024). The role of FtsH4 in this recovery is also schematically shown in **Fig. 1**.

### The FtsH1/3 complex regulates the transcriptional response to P starvation

FtsH1/3 is also involved in adaptation to P starvation through a mechanism analogous to the one allowing the adaptation to Fe deficiency. Utilization and uptake of P, activated in response to P starvation, are under the control of Pho regulon in bacteria. It involves a two-component system, which consists of a sensor kinase (PhoR) and a response regulator (PhoB) (Santos-Beneit 2015). Their action is regulated by a negative regulator (PhoU) and a phosphate-specific transport system (PstSCAB) (Su et al. 2007). Recent findings have shown that the Pho regulon response and hence the P uptake system encompassing PstS, PstS2, SphX and PhoA, is significantly downregulated in the FtsH1/3-deficient mutants (Krynická et al. 2019). Moreover, the *ftsH3* knock-down mutant exhibits a reduced level of ribosomal proteins even under nutrient sufficiency. Intriguingly, a similar pattern of depletion of ribosomal proteins was observed in WT only when it encountered P depletion, suggesting that the *ftsH3* knock-down mutant is already P-deprived, even under P-replete conditions. This repression could be caused by the accumulation of PhoU under both P-replete and P-deplete conditions in FtsH1/3-deficient mutants. These findings indicate that FtsH1/3 plays a crucial role in the modulation of the PhoU level, similar to its role in the regulation of the Fur level (**Fig. 2**). The continuous repression of the Pho regulon caused by the accumulation of PhoU compromises the mutant’s ability to effectively adapt to P deficiency.

### FtsHs contribute to the maintenance of C and N balance under stress conditions

The complexity of FtsH’s role in adapting to nutritional stress is evident from the observation that FtsH mutants exhibit a deficient transcriptional response not only to Fe and P depletion but also to C and N starvation. The metabolism of C and N must be closely coordinated. Cyanobacteria evolved a sophisticated regulatory network to maintain the cellular C/N balance. Signal transduction between the C and N pathways is mediated by the metabolite 2-oxoglutarate (2-OG), which represents the C skeleton utilized for N assimilation (Huergo and Dixon 2015). The amount of 2-OG in the cell increases when there is an excess of Ci or a deficiency of N. Conversely, it decreases markedly when there is a deficiency of Ci or an excess of N. The level of 2-OG is sensed by several regulatory proteins involved in C/N metabolism (Forchhammer and Selim 2020).

#### Ci uptake.

For maintaining sufficient levels of Ci, cyanobacteria possess a refined CO_2_-concentrating mechanism (CCM) that is triggered by low Ci availability (Kaplan et al. 1999). The CCM involves a set of proteins and their complexes like Ndh-1S, SbtA or CmpA, which facilitate cells to acquire Ci from the environment. The expression of these proteins is under the control of the NdhR transcription repressor (Klahn et al. 2015). At a high Ci level, the binding of abundant 2-OG to NdhR enhances the affinity of NdhR for DNA and causes subsequent repression of genes for Ci transporters. Conversely, under Ci deficiency, when the level of 2-OG is low and 2-phosphoglycolate is elevated, NdhR is released from DNA to the cytoplasm permitting expression of the NdhR regulon (Burnap et al. 2015, Hagemann et al. 2021). Alike- the previously mentioned requirement of FtsH2/3 for the expression of NdhR regulon induced upon the shift to low Ci (Zhang et al. 2007, Klahn et al. 2015; **Fig. 1**), the downregulation of FtsH1/3 also led to a similar effect (Krynická et al. 2019). Conversely, an excess of FtsH1 resulted in the induction of the NdhR regulon and the accumulation of CCM proteins even under conditions of Ci sufficiency (Krynická et al. 2019). These findings suggest that both FtsH2/3 and FtsH1/3 play a critical role in the efficient induction of NdhR regulon. The accumulation of the NdhR repressor in mutants with a reduced level of FtsH1/3 under low Ci conditions indicates that similar to Fur and PhoU, NdhR might be a target for FtsH-mediated degradation. Although FtsH1/3 primarily resides in PM, while FtsH2/3 in TM, both complexes are oriented toward the cytoplasm (**Fig. 1**). This suggests that like in the case of Fur, the fraction of NdhR released from DNA to the cytoplasm is preferentially degraded due to Ci-limiting conditions. Moreover, a recent report indicates that the third cyanobacterial FtsH complex, FtsH4, also contributes to the regulation of C metabolism. Several proteins associated with the CCM have been identified as potential substrates of FtsH4 (Koník et al. 2024), and it is possible that upon reaching a sufficient concentration of carbon dioxide in the cell, FtsH4 reduces the amount of Ci transporters to maintain the balance between C and N (**Fig. 1**).

#### N uptake.

The global transcription regulator NtcA plays a crucial role in maintaining N homeostasis (Espinosa et al. 2007, Llácer et al. 2010). Under N sufficiency, NtcA binding to DNA is limited due to the low concentration of 2-OG, which binds to NtcA and stimulates the NtcA regulon response. Furthermore, the PII protein forms a complex with PII-Interacting Protein X (PipX), which otherwise binds to NtcA and co-activates it (Espinosa et al. 2014). P_II_ is a signal transduction protein that assesses and integrates the current C/N/energy status of cells via the interdependent binding of ATP, ADP and 2-OG (Fokina et al. 2011, Forchhammer and Selim 2020). N starvation, accompanied by a rise in the C/N ratio, results in the accumulation of 2-OG, which, in turn, stimulates the DNA binding of NtcA. 2-OG also binds to P_II_, which undergoes phosphorylation, leading to the release of PipX from P_II_ binding. This enables the formation of the NtcA/2-OG/PipX ternary complex, enhancing the affinity of NtcA for a set of promoters inducing the expression of the genes needed for N uptake (Osanai and Tanaka 2007, Forchhammer et al. 2022). A detailed schematic representation of the NtcA regulon control is provided in **Fig. 3**. Mutants with a reduced level of FtsH1/3 displayed problems with the induction of the NtcA regulon under N limitation. However, in this case, no negative regulator as a potential substrate for FtsH degradation has yet been identified. On the other hand, both FtsH1- and FtsH3-depleted mutants, deficient in FtsH1/3, encounter difficulties in the phosphorylation of the P_II_ protein. A potential explanation could be that the insufficient phosphorylation of the P_II_ protein is attributed to the accumulation of P_II_ phosphatase PphA (Kloft and Forchhammer 2005), which could potentially serve as a substrate for FtsH1/3. However, this hypothesis has been ruled out, as the amount of PphA in the FtsH1/3 knock-down mutant was comparable to that of WT (Krynická et al. 2019). Although we do not know what causes the lack of PII phosphorylation, it results in the persistent suppression of the NtcA regulon in the FtsH1/3-deficient mutants (**Fig. 3**). Thus, the precise mechanism of this FtsH1/3-mediated regulation remains unknown.

### FtsH protease is involved in osmoregulation in *Synechocystis*

The role of FtsH2/3 in osmoregulation was discovered through a screen for mutants of *Synechocystis* with increased tolerance to nonionic osmotic stress (Stirnberg et al. 2007). The growth of the *ftsH2*-null mutant under high salinity conditions was severely impaired due to reduced levels of the compatible osmoprotectant glucosylglycerol (GG), despite normal transcription of the *ggpS* gene encoding the GG-phosphate synthase (GgpS). In cells with deleted *ftsH2*, the GgpS content increased, but its synthase activity remained low. The low level of GG led to a reduced salt tolerance in the *ftsH2*-null mutant, which could be restored by adding external osmolytes. An in vitro assay using inverted membrane vesicles demonstrated the proteolytic degradation of GgpS by FtsH2. GgpS is a part of a GG synthesizing complex, as indicated by its interaction with GG-phosphate phosphatase in yeast two-hybrid screens. Stirnberg et al. (2007) identified GgpS as the first soluble substrate of a cyanobacterial FtsH protease. It suggested that the salt-sensitive phenotype of the *ftsH2*-null mutant is the consequence of inefficient removal of the inactive GgpS enzyme, which cannot synthesize GG, the main osmoprotective solute of *Synechocystis.*

In summary, FtsHs and especially FtsH1/3 play a crucial role in the regulation of gene expression under various nutrient stress conditions, including Fe, P, Ci and N limitation. Cells benefit from a strategy where a protease complex is involved in the regulation of abundance of several transcription factors. The protease most probably degrades just factors that are DNA-free since it is not able to reach (due to different localization of bound and unbound factors) or, more probably, to recognize (due to different conformations) DNA-bound transcription factors associated with the corresponding nutrient, which increases their affinity for DNA. In this way, especially the FtsH1/3 complex functions as a key epistatic facilitator, bridging various regulatory pathways and fine-tuning the transcriptional nutrient stress response in *Synechocystis* (Krynická et al. 2019). Beyond the transcriptional control during starvation, FtsHs are also responsible for the removal of the inactive enzyme, which plays the important role in resistance to osmotic stress, and stress-induced proteins like IsiA or CCM proteins. Such scavenging of redundant stress-specific proteins contributes to the fast post-stress restoration and maintenance of the nutrient balance. This role has been identified for FtsH2/3 and FtsH4, but it cannot be excluded that FtsH1/3 serves a similar function.

## FtsH Proteases Play a Critical Role in Light Adaptation

### The role of FtsH2/3 in the maintenance of the functional PSII complex during high light and UV stress

**Fig. 4:**
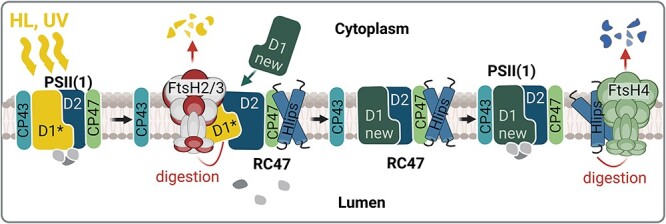
The role of the thylakoid FtsHs in photoprotection of the PSII complex. Under HL and UV stress, the fast inactivation of PSII is accompanied by the damage of the D1 protein (D1*) leading to the destabilization of CP43 binding within PSII. This destabilization allows access of FtsH2/3 to the damaged D1, which is then quickly degraded and replaced by the newly synthesized D1 (new D1). Subsequently, the CP43 antenna is fully reconnected to the repaired RC47. This repair of PSII occurs in the presence of high-light inducible proteins (Hlips), which protect PSII intermediates from oxidative damage by quenching the energy from the excited Chl. When the PSII repair is completed, the Hlips are released from the complex and their level is regulated by the FtsH4 protease. Created using BioRender.com.

FtsHs play a crucial role in the adaptation to high light (HL) and UV stress in all photosynthetic organisms [reviewed by Kato and Sakamoto (2018)]. They preserve the integrity and functionality of the photosynthetic apparatus under stress conditions by quality control of PSII. During HL and UV stress, the D1 subunit of PSII is quickly damaged and if the rate of this damage exceeds the rate of PSII repair, the loss of photosynthetic performance called photoinhibition occurs (Silva et al. 2003, Yamamoto et al. 2008, Nixon et al. 2010). In *Synechocystis*, FtsH2/3 is responsible for selective degradation of D1 during the fast PSII repair (Silva et al. 2003, Komenda et al. 2006) and it also regulates levels of unassembled PSII subunits D2 and CP47 as well as RC47, a CP43-less PSII assembly intermediate (Komenda et al. 2006). Deletion of the *ftsH2* gene in *Synechocystis* resulting in a lack of FtsH2/3 increases the light sensitivity of the strains, which become chronically photoinhibited (Komenda et al. 2006, Boehm et al. 2012). However, the precise mechanism by which FtsH2/3 distinguishes between damaged and undamaged D1 subunits remains elusive. It has been proposed that the changed conformation/partial disassembly of PSII, possibly triggered by photodamage, facilitates contact between FtsH and D1 (Krynická et al. 2015). Specifically, the destabilization in the binding of CP43, which normally shields the D1 subunit, contributes to the recognition and degradation of damaged D1 (Krynická et al. 2015). It can be speculated that this detachment exposes specific regions of D1 that are recognized by FtsH2/3 in analogy to membrane protein substrates of FtsH in *E. coli* (Ito and Akiyama 2005). Kato et al. (2023) recently showed that at least in chloroplasts of *Chlamydomonas*, the effective degradation of D1 requires oxidation of the Trp14 residue at the N-terminus of D1, where the processive degradation of D1 starts (Komenda et al. 2007). The molecular dynamics data in this work indicate that the Trp oxidation increases fluctuation of the first stromal α-helix of D1, possibly allowing its enhanced interaction with FtsH2/3. The role of FtsHs in PSII repair is shown schematically in **Fig. 4**. It remains to be determined whether FtsH2/3 can actively contribute to the CP43 detachment. If the CP47 antenna is absent, which increases accessibility of the D2 subunit, the FtsH2/3-mediated degradation of D2 exceeds that of D1 and occurs even in the dark. This observation indicates that protease accessibility induced by PSII disassembly, not necessarily related to photodamage, plays a crucial role in the selection of D1 and D2 subunits for degradation by FtsH. Interestingly, FtsH2/3 also participates in repairing PSII after heat stress damage (Yamamoto et al. 2008). It is probable that in this case, the recognition is again related to changes in the CP43 conformation induced by inactivation of the oxygen-evolving complex, the most heat-susceptible part of PSII (Nash et al. 1985). It is noteworthy that CP47 and CP43 are resistant to degradation in the dark (Krynická et al. 2015). Notably, although FtsH2/3 plays a major role in the degradation of both precursor and mature forms of D1 incorporated in the larger PSII complexes containing at least D2 and CP47, the unassembled D1 (e.g. in the mutant lacking D2) can be efficiently degraded even in the absence of FtsH2/3, pointing to the involvement of other protease(s) (Komenda et al. 2010).

In addition to PSII repair, the *Chlamydomonas* complex homologous to FtsH2/3 has also been shown to contribute to the turnover of subunits of cytochrome b_6_f complexes under HL conditions (Malnoë et al. 2014). This helps maintain an efficient electron transport chain and optimizes light energy utilization. Light-induced degradation of an unassembled Rieske FeS protein by an FtsH protease has also been observed in plant chloroplasts (Adam et al. 1998). Considering cyanobacteria’s ancestral relationship to chloroplasts, it is likely that similar regulatory mechanisms are present in cyanobacteria. This has recently been supported by the fact that the PetD subunit of cytochrome b_6_f was identified as a potential substrate of the FtsH4 protease in *Synechocystis* (Koník et al. 2024). PetD binds a Chl molecule (Kurisu et al. 2003, Stroebel et al. 2003), and its light-induced damage may trigger the degradation in analogy to D1 in PSII.

### FtsH4 facilitates swift acclimation to sudden changes in light conditions by regulating photosystem biogenesis

Although FtsH4 is not required for PSII repair, it has recently been shown to facilitate adaptation to changes in the light conditions (Krynická et al. 2023). Unlike FtsH2, deletion of FtsH4 has no obvious negative impact on *Synechocystis* growth under normal growth conditions (Mann et al. 2000). However, the ∆*ftsH4* mutant exhibits severely retarded growth under HL stress, especially after dilution of cells, which reached the stationary phase.

To protect the cell from excessive light absorption, the phycobilisome antennas and PSI undergo a reduction process initiated at the transcript level shortly after exposure to HL. This reduction is also accompanied by the upregulation of proteins important for HL resistance such as FtsH2, D1 and HL-inducible proteins (Hlips). Hlips are small, single-helix Chl-binding proteins important for photoprotection of PSII during biogenesis under stress conditions [reviewed in Komenda and Sobotka (2016)]. This global regulatory process is governed by the Hik33/RpaB two-component system (Los et al. 2010, Wilde and Hihara 2016). Recent research has unveiled that FtsH4 affects the response of the Hik33/RpaB-mediated regulatory pathway to HL exposure (Rachedi et al. 2020, Krynická et al. 2023) although the precise mechanism of FtsH4 action remains unclear. RpaB serves as a transcription factor that positively regulates the expression of PSI subunits and negatively influences the expression of light-inducible genes under low-light conditions (Riediger et al. 2019). Therefore, hypothetically, it might be a target for FtsH4-mediated degradation.

**Fig. 5:**
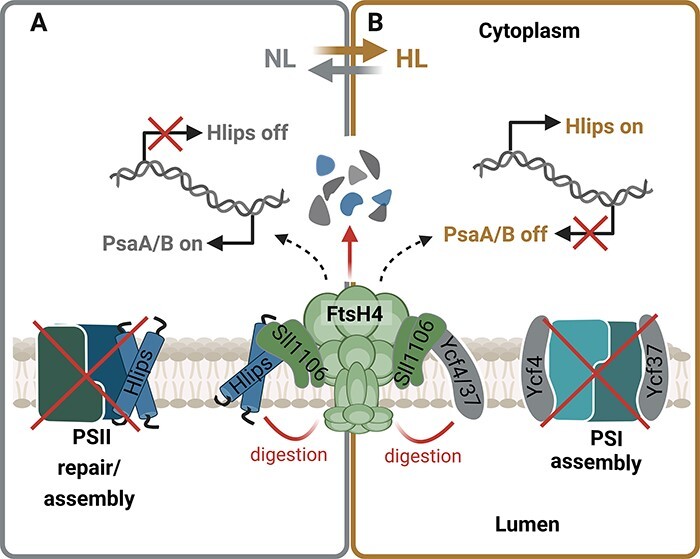
The role of FtsH4 in the fast acclimation to sudden light changes by regulating biogenesis of photosystems. (A) Upon shift to HL, FtsH4 promotes the expression of high-light inducible proteins (Hlips) important for the protection of PSII biogenesis. At the same time, it reduces the level of PSI assembly factors Ycf37 and Ycf4, which leads to a reduction of PSI biosynthesis. (B) After the shift from HL to NL, the FtsH4 reduces the level of redundant Hlips to speed up the assembly process of PSII and maximize the photosynthetic performance. Concurrently, it promotes expression of PSI subunits. This synchronized control helps the cell quickly respond to sudden changes in light conditions and contributes to the long-term adjustment of the PSII/PSI ratio. Created using BioRender.com.

In addition to transcriptional control, FtsH4 is involved in the post-stress removal of Hlip proteins once they are no longer needed (Krynická et al. 2023). This typically occurs when cells are shifted from HL back to NL, after acclimation to HL stress or during the stationary growth phase. Besides Hlips, FtsH4 also controls the level of PSI assembly factors Ycf37 and Ycf4 during HL stress (Koník et al. 2024). FtsH4 is involved in the degradation of these factors and potentially may degrade both large PSI core subunits in the unassembled state, limiting the biogenesis of new PSI complexes under conditions where its rapid arrest is needed (Koník et al. 2024). Hereby, FtsH4 regulates the biogenesis of both photosystems according to the current need to promptly respond to sudden light changes. Upon shift to HL, it promotes Hlips expression important for photoprotection during the PSII biogenesis. At the same time, it reduces the level of PSI assembly factors, which leads to the reduction of PSI biosynthesis. On the other hand, after moving back to NL, FtsH4 eliminates the redundant Hlips to speed up the assembly process of PSII and maximize the photosynthetic performance. Concurrently, it promotes the expression of PSI subunits. This synchronized control helps the cell quickly respond to sudden changes in light conditions and also contributes to the long-term adjustment of the PSII/PSI ratio. A schematic presentation of the role of FtsH4 in regulating the biosynthesis of photosystems is shown in **Fig. 5**. In cyanobacteria, the adjustment of photosystem stoichiometry is reached by variation of the PSI content, while the PSII level is quite stable. This is achieved by regulation at both the transcriptional level via induction of genes encoding the large Chl-binding subunits PsaA and PsaB (Muramatsu and Hihara 2003) and the post-transcriptional level via Chl availability (Kopečná et al. 2012). However, FtsH2/3 is also needed for efficient synthesis of PSI since deletion of the *ftsH2* gene causes a reduction in PSI level and Chl content of the cell (Mann et al. 2000) although the underlying mechanism is unknown. In cyanobacteria, the half-life of Chl is longer in comparison with Chl-binding proteins, indicating that Chl released from degraded proteins can be reused (Vavilin et al. 2007). It is, therefore, possible that Chl released during the D1 degradation can be partly reused for the synthesis of PSI. When D1 turnover is disrupted in the *ftsH2*-null mutant, no source of recycled Chl is available for PSI synthesis, which negatively affects the total amount of PSI in the cell. Nevertheless, since FtsHs can also function as a chaperone (Maziak et al. 2021), another possibility is that the chaperone and not the protease activity of FtsH2/3 is essential for the biogenesis of PSI. Thus, further research is needed to fully understand the molecular mechanisms by which FtsH2/3 regulates the level of PSI.

## Level and Activity of FtsH Complexes in the Cell and Their Stress-Induced Regulation

A recent study estimated the absolute cellular levels of all FtsH subunits and enabled direct comparison of their levels and the levels of key photosynthetic proteins in *Synechocystis* (Jackson et al. 2023). The abundance of FtsH1 is less than 1,000 copies per cell (cpc), which is comparable with the cell abundance of the single FtsH complex of *E. coli*. This is reported to be about 660 cpc (Wiśniewski and Rakus 2014), confirming that FtsH1 belongs to the typical bacterial FtsH isoforms located in PM (Tomoyasu et al. 1995). The three- to six-fold higher levels of FtsH2, FtsH3 and FtsH4 at 1,800–5,150, 2,400–3,000 and 2,800–5,950 cpc, respectively, may reflect their presence in the more abundant TM and functioning that extends beyond the remit of a basic bacterial FtsH protease and targets thylakoid proteins and their complexes (Jackson et al. 2023). These thylakoid protease complexes are about 10–40 fold less abundant in comparison to the PSI or PSII core subunits. Additionally, using the label-free intensity-based absolute quantification method, previously validated as equivalent to the molar amount (Schwanhäusser et al. 2011), the FtsH4 abundance scores were determined in comparison with both photosystems. Interestingly, FtsH4 was approximately 50-fold less abundant than PSI components PsaA and PsaB and 20–35 times less abundant than PSII components D1 and D2. Given the prevailing occurrence of FtsH4 as a hexamer, PSI as a trimer and PSII as a dimer, one FtsH4 complex corresponds to 60–100 PSII complexes and 100 PSI complexes (Krynická et al. 2023). The level of FtsH2/3 might be slightly higher according to cpc since the hexamer is composed of two subunits present in the 1:1 ratio (Boehm et al. 2012).

Notably, the cellular content of FtsH proteases changes in response to various stresses, thereby enhancing stress-responsive mechanisms. Generally speaking, FtsH proteins are classified as stress-inducible proteins, and their expression levels elevate upon exposure to stress, as evidenced by a comprehensive transcriptomic analysis of the *Synechocystis* genome conducted under various stress conditions (Kopf et al. 2014). Nevertheless, limited information is available concerning the mechanisms regulating their expression. The *ftsH1, ftsH2* and *ftsH3* transcripts have been recently found to be regulated by the Hik33/RpaB regulatory pathway (Riediger et al. 2019), and this explains the upregulation of their expression in response to HL and low temperature and downregulation in the darkness and stationary phase (Rachedi et al. 2020). Furthermore, the expression of *ftsH1* experiences a dramatic increase under conditions of N and Fe limitation. This observation aligns with the fact that FtsH1/3 is involved in the acclimation to such stressors, although the specific regulatory pathway responsible for this control remains unclear. In contrast to ftsH1-3, the f*tsH4* transcript does not appear to be a target for the RpaB. Instead, Hik33/RpaB response appears to be a subject of regulation by FtsH4. Notably, the expression of the f*tsH4* gene markedly increases during periods of darkness and the stationary phase. Although FtsH4 has been demonstrated to affect the transition to the stationary phase (Krynická et al. 2023), the precise role of FtsH4 in this transition remains enigmatic.

Analyses of FtsH levels under various environmental conditions are scarce. Krynická et al. (2014) showed that the level of all FtsHs increases in response to Fe depletion. In accordance with the transcript increase, the levels of FtsH2 and FtsH3 increase in response to HL and the FtsH4 level increases in the stationary phase with its peak in the linear growth phase (Koník et al. 2024). The question also remains what regulates the level of FtsH proteins in the cell. A recent study has shown that FtsH4 exhibits a high auto-degradation activity (Krynická et al. 2023). It is, therefore, possible that this protease is a subject of autoproteolytic control in vivo. Substrate trapping has also revealed FtsH3 as a putative substrate for FtsH4. FtsH2/3 is involved in the repair of PSII during HL stress and needs to be present in the vicinity of PSII complexes. Given the high turnover indicated by the radioactive labeling of FtsH2 and FtsH3 observed during the HL exposition (Linhartová et al. 2014, Krynická et al. 2023), FtsH2/3 is likely prone to HL-induced damage too. Thus, FtsH4, which is probably localized at the site of repair and biosynthesis of new photosynthetic complexes similar to FtsH2/3 (Knoppová et al. 2022), might be responsible for the quality control of FtsH2/3. Two studies on cyanobacterial FtsH, not focused on *Synechocystis*, investigated the expression of *ftsH* genes in various ecotypes of *Prochlorococcus* picocyanobacteria (Gómez-Baena et al. 2009, Bonisteel et al. 2018). The first study conducted on low-light-adapted *Prochlorococcus marinus* strains SS120 shows that FtsH1 and FtsH2 (referred to as FtsH2 and FtsH3, respectively, in *Prochlorococcus*) specifically responded to oxidative damage induced by Dichorophenyl-dimethylurea and 2,5-dibromo-3-methyl-6-isopropylbenzoquinone treatment and N starvation, highlighting their role in the redox response and acclimation to N limitation in this strain (Gómez-Baena et al. 2009). A subsequent study of two additional *Prochlorococcus marinus* strains, the HL-adapted MED4 and the low-light-adapted MIT 9313, further supported the involvement of the FtsH2/3-like complex in PSII repair. The MED4 and MIT 9313 strains exhibit significant differences in their capacity to remove the D1 protein during the PSII repair process. High-light-adapted MED4 demonstrates inducible expression of isoforms homologous to FtsH2 and FtsH3, consistent with its faster D1 removal rate. In contrast, the low-light-adapted MIT 9313 is unable to rapidly induce the expression of FtsH2 and FtsH3 homologs, thereby limiting its ability to remove damaged D1 from the reaction center. The predominance of the FtsH4 isoform in the PSI-rich MIT 9313 is in agreement with the involvement of FtsH4 homohexamers in the PSI assembly and maintenance, rather than the direct participation in PSII repair (Bonisteel et al. 2018).

The level of FtsH2/3 can be indirectly affected by regulating the abundance of Psb29. Psb29 was originally considered an assembly factor of PSII (Keren et al. 2005), but the later study unequivocally showed its physical interaction with FtsH2/3 and depletion of FtsH2/3 in the Psb29 absence (Bečková et al. 2017a). Since the synthesis of FtsH2/3 remained unaffected in the *psb29*-null mutant, the interaction-mediated stabilization of FtsH2/3 is the most probable mechanism of the Psb29 action. FtsH2/3 has also been co-isolated with the thylakoid prohibitin, a member of the band 7 protein family (Boehm et al. 2009), which is known to regulate the activity of FtsH in *E. coli* (Kihara et al. 1996). Interestingly, deletion of four existing genes for band 7 proteins including this prohibitin, putative stomatin and flotillin did not show any apparent effect on the physiology and light sensitivity of *Synechocystis* (Boehm et al. 2009) raising questions about the function of this protein family in cyanobacteria. Similarly, the amount of FtsH4 could be regulated by the Sll1106 protein, which was recently discovered as a binding partner of FtsH4 (Krynická et al. 2023; **Fig. 5**). We cannot exclude a role of other protein factors like prolyl-*cis*-*trans* isomerases in the FtsH2/3 stability as immunophilin CYN28 with this activity is required for FtsH1/2 accumulation upon HL stress in *Chlamydomonas* (Fu et al. 2023).

The substrate specificity and activity of FtsH1/3 should be restricted to proteins present within or in the vicinity of PM (Krynická et al. 2014, Sacharz et al. 2015). However, its ability to degrade transcription factors seemingly contradicts this assumption since the main localization of cyanobacterial DNA is the central cytoplasmic cavity apparently separated from PM by TMs and transcription factors are expected to be localized there too. Thus, either these factors are degraded when detached from DNA and diffuse to PM or some DNA reaches PM, and transcription factors are degraded there. Given the existing coupling between transcription and translation in prokaryotes (Weixlbaumer et al. 2021) and the need for co-translational insertion of PM integral proteins, the proximity of some DNA regions to PM is possible. Some DNA loops containing genes for PM proteins can reach PM either at the thylakoid convergence zones (Rast et al. 2019) or through perforations observed in cyanobacterial thylakoids using electron microscope tomography (Nevo et al. 2007). Such a scenario would also be in agreement with the DNA-position-dependent expression of genes observed in *E. coli* (Bryant et al. 2014). Concerning the thylakoid FtsH complexes, the patchy distribution of thylakoid FtsH2/3 and especially FtsH4 suggests their action in limited areas of TM (Krynická et al. 2014, 2023, Sacharz et al. 2015). Specifically, FtsH4 spots at the periphery of TM and their action on PSII and PSI assembly factors (Krynická et al. 2023, Koník et al. 2024) suggest its very specific localization within biogenic thylakoid regions close to PM called thylapses (Rast et al. 2019). Such centers could be an analogy to *Chlamydomonas* translation zones near the pyrenoid (Uniacke and Zerges 2007). On the other hand, somewhat more diffuse patches of FtsH2/3 may mark its more widespread occurrence within thylakoid regions, which could be, for instance, the above-mentioned perforations inside the membrane system that are occupied with ribosomes (Nevo et al. 2007) and the selective D1 replacement could take place there.

Finally, the phosphorylation of FtsH1 and FtsH2 in algal chloroplasts affecting its function (Kato and Sakamoto 2019) indicates that FtsH activity can also be regulated by post-translational modifications. Similar regulation of cyanobacterial FtsHs has not yet been described; however, phosphorylation at Ser84 of the FtsH2 homolog in *Synechocystis* has been found (Spät et al. 2015).

## Prospects for Future Research

There are a number of fundamental questions concerning the function of FtsH complexes in general and specifically in cyanobacteria (and chloroplasts) awaiting answers. The detailed atomic structures of cyanobacterial FtsH complexes remain to be solved in order to see the interaction and interplay between individual subunits, especially in heterocomplexes. Their spatial location and arrangement together with neighbor complexes, as revealed for the FtsH complex with HlfKC in *E. coli* (Qiao et al. 2022) using cryo-electron microscopy, would also help elucidate the regulatory and specificity aspects of the complexes. In fact, the specific location and protein environment can also explain the substrate specificity, which is different for each cyanobacterial FtsH complex, and the reason for it needs to be elucidated.

As concerns the individual complexes, the obvious unresolved issue is how the FtsH3 subunit is targeted to both PM, where it forms the heterocomplex with FtsH1, and TM, where it forms the heterocomplex with FtsH2. Data of Mahbub et al. (2020) may suggest that the location of the individual membrane proteins is given by targeting their mRNA by specific membrane-bound mRNA-binding proteins. Thus, a protein specifically binding to the *ftsH3* transcript could be located in both PM and TM, while these proteins specific for the *ftsH1, ftsH2* and *ftsH4* transcripts could bind just to one membrane type.

FtsH1/3 localized in PM is involved in the acclimation to nutrient stress. It controls the level of negative transcriptional regulators released from complexes with DNA or other regulatory proteins upon the onset of the stress. The main unanswered question concerning this protease is what is the reason for its indispensability in *Synechocystis* (and most probably also in other cyanobacteria). Further studies devoted to functions previously identified in *E. coli* (for instance, cell division and synthesis of lipids and lipopolysaccharides) are needed to answer this question. Since FtsH1/3 appears to directly degrade transcription factors, it is not clear whether it targets these proteins whenever they are not bound to DNA or whether their recognition is also dependent on their specific conformation affected by binding with ligands (for instance, nutrients).

The thylakoid FtsH2/3 complex primarily performs the quality control of PSII as it is responsible for the degradation of D1 during its selective replacement and for the removal of the unassembled PSII subunits. Although the accessibility of D1 and D2 was shown to be the crucial factor, it is not quite clear which specific parts of these proteins allow their recognition and subsequent degradation. The role of small PSII subunits attached to these large proteins cannot be excluded. FtsH2/3 also affects the abundance of PSI via the so far unknown mechanism, and elucidation of this mechanism and possible involvement of the ATPase chaperone domain also remain a task for future research. FtsH2/3 contributes to acclimation to low Ci stress by regulating the expression of CO_2_/bicarbonate transporters, and the interconnection of this regulation with the FtsH1/3-mediated control of CO_2_/bicarbonate uptake deserves further attention.

The second thylakoid FtsH4 complex controls the biogenesis of both PSII and PSI in response to light changes. Regulation occurs at both the transcript and protein levels through the degradation of redundant transcription regulators and assembly factors, respectively. Since FtsH4 is often co-isolated with FtsH2/3 and PSII complexes (Knoppová et al. 2022), the possible overlap in the location of these complexes within TM needs to be clarified. The mass spectrometry–derived study of FtsH4 substrates also indicates a possible role of FtsH4 in CO_2_/bicarbonate transport, and a more detailed view on this aspect is needed.

## Data Availability

The data underlying this article are available in the article.
